# Analysis and enhancement of risk management for ethnic differences in antineoplastic drugs in Japan

**DOI:** 10.1186/s12913-022-08685-w

**Published:** 2022-10-26

**Authors:** Shinobu Uzu, Jun Sato, Rika Wakao, Takahiro Nonaka

**Affiliations:** 1 Pharmaceuticals and Medical Devices Agency, Tokyo, 100-0013 Japan; 2 Office of New Drug V, Tokyo, Japan; 3 Office of Research Promotion, Pharmaceuticals and Medical Devices Agency, Tokyo, 100-0013 Japan; 4 Osaka City University, Osaka, Japan

**Keywords:** Drug safety, Labeling, Ethnicity, Drug development, Risk management plan

## Abstract

**Background:**

Risk management in the post-marketing phase is crucial to minimize health problems caused by drugs. Because ethnic factors may affect drug safety, the objective of this study was to explore concrete approaches to reflecting ethnic factors in risk management under multi-regional drug development.

**Methods:**

We assessed Pharmaceuticals and Medical Devices Agency (PMDA) review reports on antineoplastic drugs approved as new molecular entities in the last 10 years to identify any differences in the incidence of adverse drug reactions (ADRs) related to myelosuppression, hepatic impairment, renal impairment, and interstitial lung disease between Japanese and non-Japanese populations. In addition, we investigated how those ADRs were handled in the labeling of each drug.

**Results:**

In total, 44 drugs were available for comparing the incidence of ADRs between Japanese and non-Japanese populations. Of these, 32 drugs had a higher incidence of ADRs in the Japanese population. However, the incidence of ADRs in the Japanese population was described in the labeling for 7 drugs, and only the incidence in the overall population in multi-regional phase III trials was described in the labeling for the remaining 25 drugs. Of these 25 drugs, two drugs were immediately placed under emergency safety control measures after approval because of the high incidence of ADRs in Japanese patients.

**Conclusions:**

For drugs that might cause serious ADRs and with a higher incidence in the Japanese population, information should be provided on the incidence in the Japanese population as well as in the overall population.

## Background

**Fig. 1:**
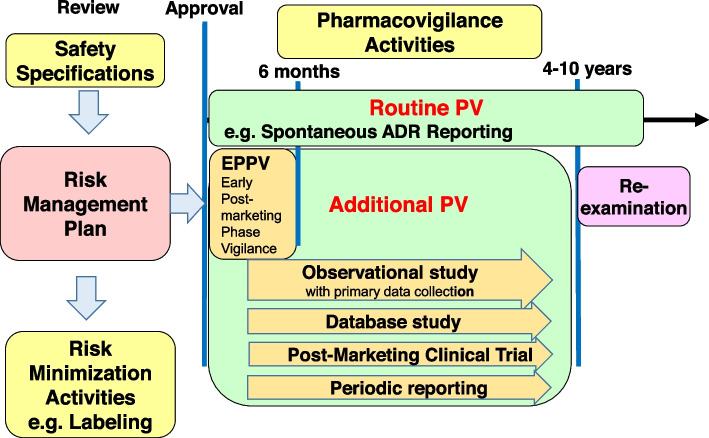
Overview of risk management plan in Japan

Because clinical trials in the drug development stage are subject to various limitations, such as the age and concomitant therapies of the subjects, there are relatively few safety databases available at the time of approval [1]. Therefore, risk management at the post-marketing phase is extremely important to ensure the safety of drugs. Similar to the risk elimination and mitigation strategies in the U.S. and the risk management plans in the EU, Japan has enforced a risk management plan [2], which allows for systematic implementation of risk minimization activities such as precautions in the labeling, and pharmacovigilance (PV) activities such as early post-marketing phase vigilance (EPPV), and observational studies with primary data collection (Fig. 1).

Regulatory authorities make an effort to find signals of health problems in the pre-marketing phase with the aim of minimizing safety issues in the post-marketing phase. In the U.S., "accelerated approval" and "boxed warnings" were identified as pre-marketing factors to predict post-marketing safety measures such as introduction of new boxed warnings [3, 4]. Because ethnic factors may affect the safety of drugs, information on ethnic differences appear to also contribute to risk minimization. In fact, in the case of Gefitinib, the incidence of interstitial lung disease (ILD) has been reported to be higher in Japan than overseas [5, 6]. The drug was launched in July 2004; in October of the same year, a revised version of the labeling, "the Dear Healthcare Professional Letters of Emergent Safety Communications", was issued with a new warning about ILD.

Complete clinical data packages for a new drug application in Japan generally include data from the Japanese population. Until the 1990s, major clinical trials such as exploratory and confirmatory clinical trials were required to be conducted with Japanese subjects. However, the ICH-E5 guideline [7] and the subsequent ICH-E17 guideline [8] now allow the use of data from non-Japanese populations as long as the complete clinical data package includes data from Japanese populations as required by the guidelines. As a result, there is no longer a need to conduct clinical trials exclusively on the Japanese population. According to the ICH-E5 guideline, ethnic factors for the acceptability of foreign clinical data are classified into intrinsic factors such as body weight and genetic polymorphism of the drug metabolism and extrinsic factors such as medical practice and diagnostics. It is practically impossible to examine the impact of ethnic factors one by one for various adverse effects. However, unlike countries or regions consisting of diverse ethnic groups, the impact of ethnic factors on safety is considered to be relatively large in countries and regions such as Japan that are composed almost entirely of one ethnic group. Under these circumstances, designing risk management by taking into account ethnic differences is key to minimizing health problems caused by drugs in the post-marketing phase.

When using data from overseas trials or multi-regional clinical trials, it is a challenge to assess the detailed ethnic differences between the Japanese and non-Japanese populations to develop risk minimization activities, because the safety database for the Japanese population is smaller in multi-regional drug development than in domestic drug development [9, 10]. As for PV activities, when there are few data of the Japanese population at the time of approval to determine concrete risk-minimization activities, all-case surveillance is often required in Japan to collect and respond to post-marketing safety information quickly and reliably. However, there is debate about the scientific rationale for conducting all-case surveillance to design appropriate PV activities [11].

Because some antineoplastic drugs are associated with serious ADRs, including fatal cases, prophylactic treatment and dose reduction need to be strictly regulated. If there is a concern about ethnic differences in the incidence of ADRs, more proactive provision of safety information would help to ensure safer use of antineoplastic drugs. In Japan, it is currently not required to clearly describe the difference in the incidence of ADRs between the Japanese and non-Japanese populations in the labeling.

Therefore, we conducted this study to investigate whether more appropriate risk management, such as precautions in the labeling and post-marketing surveillance in Japan, could be implemented, taking into account the ethnic differences between the Japanese and non-Japanese populations. We assessed the PMDA review reports on antineoplastic drugs of new molecular entities that were approved between February 2010 and August 2020 based on overseas trials or multi-regional clinical trials as the confirmatory evidence for new drug review; we used this information to compare the incidence of ADRs between Japanese and non-Japanese populations and compare the safety information provided in the Japanese and U.S. labeling. Based on the results, we proposed measures to improve risk minimization and PV activities in Japan.

## Methods

### Selection of investigated antineoplastic drugs

**Fig. 2:**
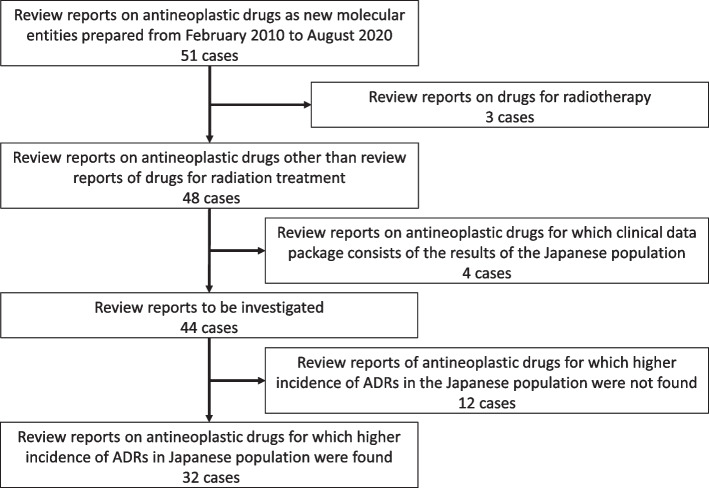
Selection process of the Pharmaceuticals and Medical Devices Agency review reports

We selected antineoplastic drugs of new molecular entities approved between February 2010 and August 2020 in Japan. The following drugs were excluded from our investigation (Fig. 2):

- i)  Drugs used for radiotherapy
- ii) Drugs for which the incidence of ADRs could not be compared between the Japanese population and non-Japanese populations in the PMDA review reports because the clinical data package for new drug applications in Japan only consisted of the results of the Japanese population. The selection of investigated antineoplastic drugs was done by J.S. and S.U. together.

### Analysis of PMDA review reports

The PMDA review reports for the selected drugs were assessed to compare the incidence of ADRs between the Japanese and non-Japanese populations and determine if there was a higher incidence of ADRs in the Japanese population. Major categories of ADRs investigated in this study were following: myelosuppression, hepatic impairment, and renal impairment, which are commonly observed with antineoplastic drugs, and ILD, for which a higher incidence is often observed in the Japanese population. A higher incidence of ADRs was defined as at least a 10% difference in the incidence for ADRs related to myelosuppression, hepatic impairment, and renal impairment, and at least a 5% difference for ILD between the Japanese and non-Japanese populations; these cutoffs were selected because they were frequently used for comparison in the review reports. The largest difference in the incidence of ADRs between the Japanese and non-Japanese populations was selected among ADRs in the same category.

For drugs with a higher incidence of ADRs in the Japanese population, the following investigations were conducted based on the information provided in the review reports:

• We classified drugs based on the mechanism of action into cytotoxic agents, tyrosine kinase inhibitors (TKIs), antibodies including antibody-drug conjugates (ADCs), immuno-oncology treatments (IOs), and hormones; then, we investigated whether there were any ADRs characteristic to the Japanese population for each category.
• We determined whether there was any relationship between the higher incidence and dosage or pharmacokinetics in the Japanese population.

The PMDA review reports were obtained from the PMDA website (https://www.pmda.go.jp/). The analysis of the PMDA review reports was conducted independently by J.S., T.N., and S.U., and conclusions were drawn through discussions by these three authors.

### Analysis of safety information in labeling

For drugs with a higher incidence of ADRs in the Japanese population, we conducted the following investigations based on the labeling:

• We compared the Japanese and U.S. labeling and examined whether the Japanese labeling contains descriptions based on a higher incidence, including descriptions only in the Japanese labeling. The comparison of the labeling between Japan and the U.S. was based on the Japanese labeling at the time the Japanese review report was finalized and the latest version of the U.S. labeling before the PMDA review report was finalized. The “Warning”, “Important Precautions” and “Serious ADRs” contents in the labeling in Japan were checked to see if they were included in corresponding parts of the labeling in the U.S.
• We investigated whether there were any drugs for which the labeling had been revised because of serious ADRs within EPPV (six months of launch). For any with revised labeling, we checked safety data related to the ADRs in the labeling of the drugs.

The labeling in Japan and the U.S. was obtained from the PMDA website (https://www.pmda.go.jp/) and the U.S. Food and Drug Administration (FDA) website (https://www.fda.gov/drugs), respectively. The analysis of the safety information in the labeling was conducted independently by T.N., R.W., and S.U., and conclusions were drawn through discussions by these three authors.

## Results

There were a total of 51 antineoplastic drugs of new molecular entities approved between February 2010 and August 2020 in Japan. Based on the exclusion criterion (Fig. 2), we selected 44 drugs for the present analysis.

### Status of drugs for which the labeling was revised because of serious ADRs in the early post-marketing phase

Intensive safety monitoring is usually conducted within six months after new drug approvals as EPPV in Japan. We searched for drugs for which the labeling related to any of the ADRs investigated in this study was revised within EPPV. This resulted in the identification of Abemaciclib and Cabazitaxel acetate. These drugs had a higher incidence of ADRs in the Japanese population than in the non-Japanese population in clinical trials. No revisions of the labeling for safety issues within EPPV were found for drugs with the similar incidence of ADRs in the Japanese and non-Japanese populations.

#### Cabazitaxel acetate

In Japan, 28 cases of severe febrile neutropenia, including five fatal cases, were reported during the first three months after its launch in September 2014. This resulted in revision of the labeling to add descriptions of antibiotic use and proper use of granulocyte colony-stimulating factor products against febrile neutropenia [12].

Cabazitaxel acetate was approved in Japan on the basis of a Japanese phase I trial and an overseas phase III trial as key clinical data. The incidence of febrile neutropenia was 54.5% (24/44 subject) in the Japanese phase I trial and 7.5% (28/371 subject) in the overseas phase III trial. The review report stated that, although the number of Japanese subjects was limited to conclude a higher incidence in the Japanese population, it is necessary to pay attention to some ADRs that seem to occur more frequently in Japanese patients [13]. The Japanese labeling included a warning on myelosuppression, as did the U.S. labeling, but it only provided the incidence of ADRs from the overseas study and did not warn of the higher incidence of febrile neutropenia in the Japanese population.

#### Abemaciclib

After the launch of Abemaciclib in November 2018 in Japan, 14 serious cases of ILD, including three fatal cases, were reported by May 2019. The Dear Healthcare Professionals Letter of Rapid Safety Communication [14] was issued in the same month, and the labeling was revised accordingly to include new warnings about ILD. According to the PMDA review report, three cases of ILD (7.9% of the Japanese subjects) were reported before the data cutoff in a pivotal trial of Abemaciclib combined with nonsteroidal aromatase inhibitor. Moreover, a case of ILD reported after the data cutoff of the trial and a case of acute pneumonia as a reported term in the ADR reporting were observed in the trial. Based on a total of five cases of ILD (13.2%) found in the clinical trial, the review report stated that the incidence of ILD tended to be higher in the Japanese population [15]. However, the labeling only described its incidence in the overall population of the multi-regional clinical trials (2.7%), but not the higher incidence (13.2%) in the Japanese population.

### ADRs with a higher incidence in the Japanese population

Of the 44 drugs eligible for this study, 32 (72.7%) had a higher incidence of one of the ADRs in the Japanese population than in non-Japanese populations (Table 1). The number of drugs with a higher incidence in the Japanese population by the ADR category was as follows: 20 drugs (45.5%) for myelosuppression, 18 drugs (40.9%) for hepatic impairment, seven drugs (15.9%) for renal impairment, and six drugs (13.6%) for ILD (Table 2). The following is a breakdown of the number of antineoplastic drugs with a high incidence (incidence rate of 50% or more) in the Japanese population, categorized by their mechanism of action. Among four cytotoxic drugs, myelosuppression was reported with all four drugs (100.0%) and hepatic impairment with three drugs (75.0%); among 22 TKIs, myelosuppression was reported with 11 drugs (50.0%) and hepatic impairment with 11 drugs (50.0%); and among seven antibodies, myelosuppression was reported with four drugs (57.1%).

**Table 1:** The list of drugs that have higher incidence of ADRs in Japanese population

Fix date of review report	Drug name	Drug category	Indication	Comparison between Japanese and non-Japanese population		Reference
				ADRs^a^	PK^b^	
Feb 2010	Panitumumab	Ab	Colorectal carcinoma	NO	Low	[16]
May 2010	Temsirolimus	TKI	Renal cell carcinoma	High	NM	[17]
Jan 2011	Eribulin mesilate	Ct	Breast cancer	High	ND	[18]
Aug 2011	Fulvestrant	Hr	Breast cancer	NO	High	[19]
Mar 2012	Crizotinib	TKI	NSCLC	High	High	[20]
May 2012	Axitinib	TKI	Renal cell carcinoma	NO	ND	[21]
Aug 2012	Pazopanib hydrochloride	TKI	Soft tissue cancer	NO	ND	[22]
Mar 2013	Regorafenib	TKI	Colorectal cancer	High	Low	[23]
Apr 2013	Pertuzumab	Ab	Breast cancer	High	ND	[24]
Aug 2013	Trastuzumab emtansin	Ab	Breast cancer	NO	ND	[25]
Oct 2013	Afatinib maleate	TKI	NSCLC	High	ND	[26]
Jan 2014	Enzalutamide	Hr	Prostate cancer	NO	ND	[27]
Apr 2014	Abiraterone acetate	Hr	Prostate cancer	High	ND	[28]
Apr 2014	Cabazitaxel acetate	Ct	Prostate cancer	High	ND	[13]
Jun 2014	Nivolumab	IO	Melanoma	High	ND	[29]
Nov 2014	Vemurafenib	TKI	Melanoma	High	ND	[30]
Mar 2015	Ramucirumab	Ab	Gastric cancer	High	ND	[31]
May 2015	Ipilimumab	IO	Melanoma	High	ND	[32]
Jul 2015	Vandetanib	TKI	Medullary thyroid cancer	High	High	[33]
Aug 2015	Trabectedin	Ct	Soft tissue tumor	High	NM	[34]
Jan 2016	Dabrafenib mesilate	TKI	Melanoma	High	NM	[35]
Feb 2016	Osimertinib mesilate	TKI	NSCLC	High	ND	[36]
Mar 2016	Ceritinib	TKI	NSCLC	High	ND	[37]
Aug 2016	Pembrolizumab	IO	Melanoma	NO	ND	[38]
Jan 2017	Afliberceot Beta	Ab	Colorectal cancer	High	NM	[39]
Jul 2017	Palbociclib	TKI	Breast cancer	High	ND	[40]
Aug 2017	Avelumab	IO	Merkel cell carcinoma	High	NM	[41]
Oct 2017	Atezolizumab	IO	NSCLC	NO	NM	[42]
Nov 2017	Olaparib	TKI	Ovarian cancer	High	ND	[43]
Apr 2018	Durvalumab	IO	NSCLC	High	Low	[44]
Jul 2018	Abemaciclib	TKI	Breast cancer	High	NM	[15]
Aug 2018	Lorlatinib	TKI	NSCLC	NO	ND	[45]
Nov 2018	Ebcorfenib/ Binimetinib	TKI	Melanoma	High	ND	[46, 47]
Nov 2018	Dacomitinib	TKI	NSCLC	NO	ND	[48]
Jan 2019	Apalutamide	Hr	Prostate cancer	NO	ND	[49]
Apr 2019	Necitumumab	Ab	Squamous NSCLC	High	ND	[50]
May 2019	Entrectinib	TKI	Solid tumors	High	ND	[51]
Nov 2019	Darolutamide	Hr	Prostate cancer	NO	ND	[52]
Jan 2020	Cabozantinib malate	TKI	Renal cell carcinoma	High	High	[53]
Feb 2020	Tepotinib hydrochloride hydrate	TKI	NSCLC	High	ND	[54]
Feb 2020	Irinotecan hydrochloride hydrate (liposome injection)	Ct	Pancreatic cancer	High	ND	[55]
Feb 2020	Trastuzumab deruxtecan	Ab	Breast cancer	High	NM	[56]
May 2020	Capmatinib hydrochloride hydrate	TKI	NSCLC	High	ND	[57]
Aug 2020	Niraparib tosilate hydrate	TKI	Ovarian cancer	High	ND	[58]

All information in this table was obtained from PMDA review reports

*Ab* Antibody, *Ct* Cytotoxic, *Hr* Hormone, *IO* Immune oncology treatment, *NSCLC* non-small cell lung cancer, *TKI* Tyrosine kinase inhibitor

^a^“High” means that ADRs with higher incidence in the Japanese population was observed and “NO” means such ADRs was not observed

^b^High: Higher in the Japanese population, Low: Lower in the Japanese population, ND: No difference, NM: PMDA didn’t mention the comparison

**Table 2:** The number of drugs with higher incidence of ADRs in the Japanese population classified according to the mechanism of action

	Myelosuppression-	ILD	Hepatic impairment	Renal impairment
Antibody (7 drugs)	4 (57.1%)	2 (28.6%)	2 (28.6%)	0 (00.0%)
Cytotoxic (4 drugs)	4 (100.0%)	0 (00.0%)	3 (75.0%)	0 (00.0%)
Hormone (5 drugs)	0 (00.0%)	0 (00.0%)	1 (20.0%)	0 (00.0%)
IO (6 drugs)	1 (16.7%)	1 (16.7%)	2 (33.3%)	0 (00.0%)
TKI (22 drugs)	11 (50.0%)	3 (13.6%)	11 (50.0%)	7 (31.8%)
Total (44 drugs)	20 (45.5%)	6 (13.6%)	18 (40.9%)	7(15.9%)

*ILD* Interstitial lung disease, *IO* Immune-oncology treatment, *TKI* Tyrosine kinase inhibitor

We then investigated the relationship between pharmacokinetics and the higher incidence of ADRs in the Japanese population. Of the 32 drugs with a higher incidence of ADRs in the Japanese population, seven drugs had no data comparing pharmacokinetics between the Japanese and non-Japanese populations in the review reports. Of the 25 drugs whose review reports provided data on the difference in pharmacokinetics between the Japanese and non-Japanese populations, three drugs (9.4%) were identified with higher pharmacokinetics in the Japanese population, 20 drugs (62.5%) had no difference, and two drugs (6.3%) had lower pharmacokinetics in the Japanese population.

Because body weight and body surface area often differ between Japanese and non-Japanese, we also investigated the relationship between dosage regimen and the higher incidence of ADRs in the Japanese population (Table 3). The dosage regimens were classified into three patterns: the same fixed doses in and outside Japan; the same body weight- or body surface area-dependent doses in and outside Japan; and different doses between the Japanese and non-Japanese populations. ADRs with a higher incidence in the Japanese population were reported with 19/29 drugs used at the same fixed doses in and outside Japan, 8/10 drugs used at the same body weight- or body surface area-dependent doses in and outside Japan, and 5/5 drugs used at different doses between the Japanese and non-Japanese populations.

**Table 3:** The relation between the number of drugs with higher incidence of ADRs in the Japanese population and dosage regimen

Dosage regimen	Higher incidence	Similar incidence	Total
Same and Fix dose	19	10	29
Same but adjusted by BW/BSA	8	2	10
Different	5	0	5

*BW* Body weight, *BSA* Body surface area

### Description of the incidence of ADRs in the Japanese population in labeling

The labeling of the following nine drugs provided precautions for ADRs only in Japan but not their actual incidence in the Japanese population. These include ILD for Pertuzumab; myelosuppression for Ramucirumab and Olaparib; liver damage for Dabrafenib mesilate, Cabozantinib malate, and Vandetanib; and renal damage for Tepotinib hydrochloride hydrate, Irinotecan hydrochloride hydrate, and Capmatinib hydrochloride hydrate (Table 4).

**Table 4:** Description of ADRs in labeling

	ADRs with higher incidence in the Japanese population	Description in labeling
Temsirolimus	ILD: JP. 11/20(55%), nJP. 52/178 (29.2%)	ND
Eribulin mesilate	Neutropenia: JP. 80/81 (98.8%), nJP. 481/827 (58.2%)	a)
Crizotinib	Neutropenia: JP. 3/15 (20%), nJP. 3/104 (2.9%)	a)
Regorafenib	ALT increased: JP. 13/65 (20%), nJP. 15/433 (3.4%)	ND
Pertuzumab	ILD: JP. 2/26 (7.7%), nJP. 7/381 (1.8%)	b)
Afatinib maleate	ILD: JP. 4/54 (7.4%), nJP. 3/175 (1.7%)	a)
Abiraterone acetate	Hepatotoxicity: JP. 20/48 (41.7%), nJP. 90/543 (16.6%)	a)
Cabazitaxel acetate	Myelosuppression: JP. 44/44 (100%), nJP. 130/371 (35%) AST increased: JP. 6/47 (12.5%), nJP. 4 (1.1%)	ND
Nivolumab	AST increased: JP. 14/52 (26.9%), nJP. 28/345 (8.1%)	a)
Vemurafenib	Hepatic impairment: JP. 5/11 (45.5%), nJP. 91/337 (27.5%)	ND
Ramucirumab	Neutropenia: JP. 58/68 (85.3%), nJP. 120/259 (27.5%)	b)
Ipilimumab	AST increased: JP. 4/20 (20%), nJP. 1/131 (0.8%)	ND
Vandetanib	ILD: JP. 1/14 (7.1%), nJP. 2/231 (0.9%) Renal impairment: JP. 5/14 (35.7%), nJP. 49/231(21.2%) Hepatic impairment: JP. 3/14 (21.4%), nJP. 29/231 (8.2%)	b) (Hepatic impairment)
Trabectedin	Neutropenia: JP. 64/73 (87.7%), nJP. (75/130 (57.7%) ALT increased: JP. 52/73 (71.2%), NJP. 72/130 (55.4%)	a)
Dabrafenib mesilate	Hepatic impairment: JP. 6/12 (50%), nJP. 39/398 (9.8) Myelosuppression: JP. 8 (66.7%), nJP. 48/398 (12.1%)	b) for hepatic impairment
Osimertinib mesilate	White blood cell count decreased: JP. 21/80 (26.3%), nJP. 10/331 (3%)	ND
Ceritinib	Blood ALP increased: JP. 11/19 (57.9%), nJP. 14/105 (13.3%) Blood creatinine increased: JP. 9/19 (47.4%), nJP. 17/105 (16.2%) White blood cell count decreased: JP. 4/19 (21.1%), nJP. 2/105 (1.9%)	ND
Afliberceot Beta	Neutropenia: JP. 46/62 (74.2%), nJP. 238/611 (39.0%)	a)
Palbociclib	White blood cell count decreased: JP. 17/27 (63.0%), nJP. 88/318 (27.7%) ALT increased: JP. 7/32 (21.9%), nJP. 37/412 (9.0%)	ND
Avelumab	Anemia: JP. 8/43 (18.6%), nJP. 105/1764 (6.0%)	ND
Olaparib	White blood cell count decreased: JP. 5/8 (62.5%), nJP. 6/187 (3.2%)	b)
Durvalumab	ILD: JP. 53/72 (73.6%), nJP. 108/403 (26.8%)	ND
Abemaciclib	Neutrophil count decreased: JP. 34/43 (79.1%), nJP. 103/277 (37.2%) ALT increased: JP. 15/43 (34.9%), nJP 24/277 (8.7%) Blood creatinine increased: JP. 13/43 (30.2%), nJP. 24/277 (8.7%)	ND
Ebcorfenib/ Binimetinib	Anemia: JP. 3/10 (30%), nJP. 50/439 (11.4%)	ND
Necitumumab	Neutrophil count decreased: JP. 53/90 (58.9%), nJP. 8/538 (1.5%) ALT increased: JP. 17/90 (18.9%), NJP 27/538 (5.0%)	ND
Entrectinib	Blood creatinine increased: JP. 11/16 (68.8%), nJP. 48/190 (25.3%) AST increased: JP. 9/16 (56.3%), nJP. 32/190 (16.8%) White blood cell count decreased: JP. 4/16 (25.0%), nJP. 9/190 (4.7%)	ND
Cabozantinib malate	Renal impairment: JP. 15/35 (42.9%), nJP. 70/331 (21.1%) Hepatic impairment: JP. 25/35 (71.4%), nJP. 93/331 (28.1%)	b) for hepatic impairment
Tepotinib hydrochloride hydrate	Blood creatinine increased: JP. 9/17 (52.9%), nJP. 22/113 (19.5%)	b)
Irinotecan hydrochloride hydrate (liposome injection)	Hepatic impairment: JP. 19/46 (41.3%), nJP. 20/117 (17.1%) Myelosuppression: JP. 38/46 (82.6%), nJP. 76/117 (65.0%)	b) for hepatic impairment
Trastuzumab deruxtecan	Neutrophil count decreased: JP. 22/30 (73.3%), nJP. 35/154 (22.7%) ILD: JP. 51/316 (16.1%), nJP. 21/329 (6.4%) AST increased: JP. 7/21 (33.3%), nJP. 2/29 (6.9%)	ND
Capmatinib hydrochcrolide hydrate	Blood creatinine increased: JP. 25/45 (55.6%), nJP. 60/289 (20.8%) ALT increased: JP. 10/45 (22.2%), nJP. 32/289 (11.1%) Platelet count decreased: 8/45 (17.8%), nJP. 6/289 (2.1%)	b) for renal impairment
Niraparib tosilate hydrate	Platelet count decreased: JP. 12/19 (63.2%), nJP. 77/367 (21.0%)	ND

*ILD* Interstitial lung disease, *JP* Japanese population, *ND* No difference in the labeling between U.S. and Japan, *nJP* non-Japanese Populations

a) The incidence of the Japanese population is described

b) A precaution for indicated ADRs is described in Japanese labeling only, but the incidence is derived from the overall population or non-Japanese population.

The labeling of only seven drugs described higher incidence of ADRs in the Japanese population. Myelosuppression was reported with four drugs, ILD with one drug, and hepatic impairment with three drugs (some drugs had two ADRs with a higher incidence in the Japanese population). For other drugs, the incidence of ADRs in the overall population of overseas confirmatory trials and multi-regional trials were described in the labeling.

## Discussion

In this study, we selected 44 antineoplastic drugs approved as new molecular entities whose review reports were prepared between February 2010 and August 2020 and examined how any differences in ADRs between the Japanese and non-Japanese populations were reflected in the labeling. We found that, although 32 drugs had a higher incidence of ADRs in the Japanese population, information on the higher incidence was not provided in the labeling for many of the drugs (25/32). It is noteworthy that, for two of them, urgent safety measures were taken soon after their approvals because of the high and serious incidence of ADRs in the Japanese population. For the 12 drugs for which the incidence of ADRs was similar in both populations, there were no revisions to the safety-related labeling in the EPPV.

In this study, antineoplastic drugs were classified into cytotoxic drugs, antibodies, TKIs, IOs, and hormones according to their mechanism of action, and the incidence of ADRs was compared between the Japanese and non-Japanese populations. We found that all four cytotoxic drugs had a higher incidence of myelosuppression in the Japanese population. Three of the four drugs showed a high incidence of hepatic impairment in the Japanese population (Table 2), and another drug, Eribulin mesilate also showed a trend toward a higher incidence of hepatic impairment in the Japanese population (7/81 subjects, 8.6%) than in non-Japanese populations (16/827 subjects, 1.9%) according to the review report [18]. Although it is difficult to interpret the data from as few as four drugs, antineoplastic drugs, which are classified as cytotoxic drugs, tend to have a higher incidence of myelosuppression and hepatic impairment in the Japanese population. However, drugs categorized as antibodies, TKIs, IOs, and hormones seem not to be associated with a consistently higher incidence of ADRs in the Japanese population.

We investigated the difference of the pharmacokinetics between the Japanese and non-Japanese populations for 25 drugs with ADRs that have a higher incidence in the Japanese population. Among them, Crizotinib, Vandetanib, and Cabozantinib malate were reported to show higher pharmacokinetics in the Japanese population, and two of them, Crizotinib and Vandetanib, had similar pharmacokinetics when corrected for body weight [20, 33, 53]. Two other drugs, Regorafenib and Durvalumab, had lower pharmacokinetics in the Japanese population [23, 44]. The other 20 drugs did not differ. Thus, specific correlation between pharmacokinetics and ADR incidence could only be found for some drugs whose high pharmacokinetics in Japanese might be responsible for the high ADR incidence. We also investigated whether dosage adjustment has an impact on the high incidence of ADRs in the Japanese population. The results showed that the percentage of drugs with high ADR incidence in the Japanese population was low for drugs used at the same fixed dose (19/29), high for drugs used at the same but adjusted dose (8/10), and high for drugs used at different doses (5/5). If weight difference has an effect on the incidence of ADRs, the fixed dose is expected to have higher incidence of ADRs in the Japanese population because Japanese people generally weigh less than Westerners. However, as noted above, the result was the opposite, suggesting no trend in terms of dosage.

Of the 32 drugs that were associated with a higher incidence of ADRs in the Japanese population, seven drugs (21.9%) provided the incidence of the Japanese population in the labeling; the rest of them did not. The incidences of ADRs in the Japanese population relative to the number of Japanese subjects in the clinical trials were as follows: neutropenia associated with Eribulin mesilate, 80/81 (98.8%); neutropenia associated with Crizotinib, 3/15 (20%); ILD associated with Afatinib maleate, 4/54 (7.4%); hepatic impairment associated with Abiraterone acetate, 20/48 (41.7%); hepatic impairment associated with Nivolumab, 12/52 (23.1%); myelosuppression associated with Trabectedin, 64/73 (87.7%); and neutropenia associated with Aflibercept beta, 46/62 (74.2%). Thus, for most of the drugs for which the incidence of the Japanese population was provided in the labeling, a relatively large number of Japanese subjects (≥ 50) was enrolled in the clinical trials. The reason for the inclusion of neutropenia as an ADR for Crizotinib in the Japanese population in the labeling is unclear, despite a relatively small number of subjects. Moreover, there were nine drugs for which the incidence of ADRs in the Japanese population was not listed but precautions for use and the incidence in the overall population were described in the labeling. It is unclear why the incidence in the Japanese population is not listed, despite the fact that Japan-specific precautions are described in the labeling. Thus, there is no criterion on how the incidence of ADRs of the Japanese population should be provided in the labeling.

The following conclusions can be drawn from the abovementioned results and discussion: (i) although 73% (32/44) of the drugs had a higher incidence of ADRs in the Japanese population than in non-Japanese populations, there was no trend toward a higher incidence of specific ADRs with specific drug categories in the Japanese population, except for some ADRs such as myelosuppression caused by cytotoxic agents; and (ii) specific correlation between pharmacokinetics and ADR incidence could only be found in some drugs whose high pharmacokinetics in Japanese might be responsible for the high ADR incidence. Accordingly, it is necessary to design risk management for each drug by determining whether ADRs are likely to occur in the Japanese population and their severity, and by considering the incidence of ADRs in clinical trials and information from other clinical trials related to the drug at the time of approval.

Regarding risk minimization activities, in particular a precaution in the labeling, the notification “Guidelines for preparing the electronic package inserts of prescription drugs” in Japan requires that ADRs that are clinically significant and need special attention should be included by taking into account the outcome and seriousness of ADRs, and that the incidence of those ADRs should be based on the results of precise and objective clinical trials [59]. Prior to the publication of the ICH-E5 guidelines in 1998, which encouraged the use of overseas clinical data in new drug applications in Japan, it was required to conduct clinical trials on Japanese subjects. As a result, labeling was prepared based on safety information for the Japanese population. In recent years, clinical data from foreign populations has been increasingly used in new antineoplastic drug applications. However, no new guidance has been published to describe safety information from Japanese populations in the labeling, and ADR incidence for Japanese populations to prevent health damage has not been provided in a uniform manner. In this study, we observed that, although the review reports for Cabazitaxel acetate and Abemaciclib showed a high incidence of serious ADRs in the Japanese population, the labeling only described the incidence in overseas or the overall population; serious ADRs occurred early in the post-marketing phase, which led to revisions of the labeling. In consideration of such cases, and in the absence of clear criteria for inclusion of the incidence of the Japanese population in the labeling, we propose that the incidence of the Japanese population should be described in the labeling by considering severity and other information related to the ADRs, even if the number of Japanese subjects enrolled in the clinical trials is too small to conduct rigorous evaluation. Such information may allow medical practitioners to take appropriate safety actions such as prophylactic treatment and dose reduction in a timely manner for the listed ADRs. For example, if the incidence of febrile neutropenia with Cabazitaxel acetate in Japanese subjects (54.5%) had been specifically alerted in the labeling, prophylactic treatment of granulocyte colony-stimulating factor should have been considered according to the guideline that requires treatment when the incidence exceeds 20% [60]. The U.S. FDA also recommends that, as much as possible, the results of clinical trials or analyses that evaluate the potential for differences in sub-groups, such as different races/ethnicities, be included in the labeling [61]. This results in more information on ethnicity provided in U.S. than European labeling [62]. For example, the Warnings and Precautions section of the U.S. labeling for Irinotecan hydrochloride hydrate (liposome dosage form) indicates that neutropenia is more common and has a higher incidence in Asians [63]. In order to enhance the description in the labeling in Japan, the next step would be to present how to include the incidence of ADRs in the Japanese population in the multi-regional drug development like the FDA guidance.

For PV activities, even in the absence of special concerns, post-marketing all-case surveillance was required for many new drugs when the number of Japanese subjects in clinical trials was small and post-marketing safety information needed to be collected quickly [11]. However, ICH-E2E guidelines requires clarification of which populations, such as patients of different racial and/or ethnic origins, have not been studied or have only been studied to a limited degree in the pre-approval phase, and that the implications of this are provided with respect to predicting the safety of the drug in the marketplace. The guideline also states that, in case no special concerns have arisen for drugs, routine PV should be sufficient for post-approval safety monitoring without the need for additional actions (e.g., safety studies) [64]. Again, in the case of Cabazitaxel acetate, post-marketing all-case surveillance contributed to the confirmation of higher incidence of febrile neutropenia in the Japanese population and the subsequent introduction of new safety measures in the early post-marketing phase. Therefore, it may be a good idea to decide on the addition of PV activities, including all-case surveillance to see the ADRs in the early post marketing phase, after clarifying the special concerns that should be investigated in the activities.

Our study has several limitations. First, the foreign population consisted of various ethnic groups but was considered one group and compared with the Japanese population, and ethnic factors such as pharmacogenomics were not taken into consideration. Second, we focused on four ADRs and compared the highest incidence of the related events. Third, the safety information in labeling was analyzed between the Warnings, Important Precautions, and Serious ADRs sections in Japan and those of Boxed Warnings, Warnings, and Precautions in the U.S. Fourth, the incidence of ADRs may reflect the impact of the adjustments of dose and/or schedules of antineoplastic drugs to reduce ADRs and the effects of combination therapies in some drugs we reviewed.

Despite the above limitations, our study provides meaningful insights from regulatory perspectives that were derived from comprehensive information rather than individual factors.

## Conclusions

For drugs that might cause serious ADRs with a higher incidence in the Japanese population than in the non-Japanese population, information on the incidence in the Japanese population should be provided. Furthermore, even if the Japanese safety database is small, additional PV activities should be converted from default all-case surveillance into tangible forms based on special safety concerns.

## Data Availability

The Japanese and the U.S. datasets analyzed during the current study are available in the PMDA website (https://www.pmda.go.jp/) and the FDA website (https://www.fda.gov/drugs).

## Abbreviations

ADCs: Antibody–drug conjugates
ADRs: Adverse drug reactions
EPPV: Early post-marketing phase vigilance
FDA: U.S. Food and Drug Administration
ILD: Interstitial lung disease
IOs: Immuno-oncology treatments
PMDA: Pharmaceuticals and Medical Devices Agency
PV: Pharmacovigilance
TKIs: Tyrosine kinase inhibitors

## References

[1] Rogers AS (1987). Drug Intelligence and Clinical Pharmacy, Vol. 21.

[2] Notification: Risk Management Plan Guidance Pharmaceutical and Medical Devices Agency Website. https://www.pmda.go.jp/files/000153333.pdf. Accessed 21 Jan 2022.

[3] Cherkaoui S, Pinnow E, Bulatao I, Day B, Kalaria M, Brajovic S, Dal Pan G (2021). The Impact of Variability in Patient Exposure During Premarket Clinical Development on Postmarket Safety Outcomes. Clin Pharmacol Ther.

[4] Schick A, Miller K, Lanthier M, Dal Pan G, Nardinelli C (2017). Evaluation of Pre-marketing Factors to Predict Post-marketing Boxed Warnings and Safety Withdrawals. Drug Saf.

[5] Kudoh S, Kato H, Nishiwaki Y, Fukuoka M, Nakata K, Ichinose Y (2008). Interstitial lung disease in Japanese patients with lung cancer: a cohort and nested case–control study. Am J Respir Crit Care Med.

[6] Iwasa E, Fujiyoshi Y, Kubota Y, Kimura R, Chandler RE, Taavola H, Norén GN, Wakao R (2020). Interstitial lung disease as an adverse drug reaction in Japan: exploration of regulatory actions as a basis for high reporting. Drug Saf..

[7] ICH guideline: E5(R1) Ethnic factors in the acceptability of foreign clinical data. ICH Website. https://database.ich.org/sites/default/files/E5_R1_Guideline.pdf. Accessed 21 Jan. 2020.

[8] ICH guideline: E17 General principles for planning and design of multi-regional clinical trials. ICH Website. https://database.ich.org/sites/default/files/E17EWG_Step4_2017_1116.pdf. Accessed 21 Jan 2022.

[9] Notification: Basic principles on Global Clinical Trials, Pharmaceutical and Medical Devices Agency Website. https://www.pmda.go.jp/files/000153265.pdf. Accessed 21 Jan 2022.

[10] Uzu S, Sekine S, Asano J, Ikuma M (2021). Assessment of the impact of Japanese-specific long-term safety data on new drug approval. Clin Transl Sci.

[11] Narkawa M (2014). Research on the Situation and Implications of the Post-marketing All-case Surveillance Study in Japan [in Japanese]. RSMP.

[12] Cabazitaxel Safety Information. Pharmaceutical and Medical Devices Agency Website. https://www.pmda.go.jp/files/000203635.pdf. Accessed 21 Jan 2022.

[13] PMDA review report: .Cabazitaxel Acetate [in Japanese] Pharmaceutical and Medical Devices Agency Website. https://www.pmda.go.jp/drugs/2014/P201400085/780069000_22600AMX00751_A100_1.pdf. Accessed 21 Jan 2022.

[14] Dear Healthcare Professionals Letter of Rapid Safety Communication: Verzenio. Pharmaceutical and Medical Devices Agency Website. https://www.pmda.go.jp/files/000229624.pdf.

[15] PMDA review report: Abemaciclib [in Japanese] Pharmaceutical and Medical Devices Agency Website. https://www.pmda.go.jp/drugs/2018/P20181004001/530471000_23000AMX00808_A100_1.pdf. Accessed 21 Jan 2022

[16] PMDA review report: Panitumumab [in Japanese] Pharmaceutical and Medical Devices Agency Website. https://www.pmda.go.jp/drugs/2010/P201000024/400256000_22200AMX00307_A100_3.pdf. Accessed 1 Aug 2022

[17] PMDA review report: Temsirolimus [in Japanese] Pharmaceutical and Medical Devices Agency Website. https://www.pmda.go.jp/drugs/2010/P201000043/67145000_22200AMX00870_A100_1.pdf. Accessed 1 Aug 2022

[18] PMDA review report:Eribulin mesilate (Initial Approval) Pharmaceutical and Medical Devices Agency Website. https://www.pmda.go.jp/files/000219170.pdf. Accessed 21 Jan 2022.

[19] PMDA review report: Fulvestrant (Initial Approval) [in Japanese] Pharmaceutical and Medical Devices Agency Website. https://www.pmda.go.jp/drugs/2011/P201100160/670227000_22300AMX01209_A100_4.pdf. Accessed 1 Aug 2022

[20] PMDA review report: Crizotinib (Initial Approval) Pharmaceutical and Medical Devices Agency Website. https://www.pmda.go.jp/files/000153949.pdf. Accessed 21 Jan 2022.

[21] PMDA review report: Axitinib [in Japanese] Pharmaceutical and Medical Devices Agency Website. https://www.pmda.go.jp/drugs/2012/P201200096/671450000_22400AMX00737_A100_1.pdf. Accessed 1 Aug 2022

[22] PMDA review report: Pazopanib Hydrochloride Pharmaceutical and Medical Devices Agency Website. https://www.pmda.go.jp/files/000153553.pdf. Accessed 1 Aug 2022

[23] PMDA review report: Regorafenib (Initial Approval) Pharmaceutical and Medical Devices Agency Website. https://www.pmda.go.jp/files/000153351.pdf. Accessed 21 Jan 2022.

[24] PMDA review report: Pertuzumab (Initial Approval) Pharmaceutical and Medical Devices Agency Website. https://www.pmda.go.jp/files/000153631.pdf. Accessed 1 Aug 2022

[25] PMDA review report: Trastuzumab Emtansin (Initial Approval) Pharmaceutical and Medical Devices Agency Website. https://www.pmda.go.jp/files/000153735.pdf. Accessed 1 Aug 2022

[26] PMDA review report: Afatinib maleate [in Japanese] Pharmaceutical and Medical Devices Agency Website. https://www.pmda.go.jp/drugs/2013/P201300171/530353000_22600AMX00017000_A100_1.pdf. Accessed 1 Aug 2022

[27] PMDA review report: Enzalutamide [in Japanese] Pharmaceutical and Medical Devices Agency Website. https://www.pmda.go.jp/drugs/2014/P201400048/800126000_22600AMX00532_A100_2.pdf. Accessed 1 Aug 2022

[28] PMDA review report: Abiraterone acetate (Initial Approval) [in Japanese] Pharmaceutical and Medical Devices Agency Website. https://www.pmda.go.jp/drugs/2014/P201400077/22600AMX00749000-A100_1.pdf. Accessed 1 Aug 2022

[29] PMDA review report: Nivolumab (Initial Approval) Pharmaceutical and Medical Devices Agency Website. https://www.pmda.go.jp/files/000209430.pdf. Accessed 1 Aug 2022

[30] PMDA review report: Vemurafenib [in Japanese] Pharmaceutical and Medical Devices Agency Website. https://www.pmda.go.jp/drugs/2014/P201400179/450045000_22600AMX01406_A100_1.pdf. Accessed 1 Aug 2022

[31] PMDA review report: Ramucirumab (Initial Approval) [in Japanese] Pharmaceutical and Medical Devices Agency Website. https://www.pmda.go.jp/drugs/2015/P201500028/530471000_22700AMX00664_A100_1.pdf. Accessed 1 Aug 2022

[32] PMDA review report: Ipilimumab (Initial Approval) Pharmaceutical and Medical Devices Agency Website. https://www.pmda.go.jp/files/000215223.pdf. Accessed 1 Aug 2022

[33] PMDA review report:Vandetanib [in Japanese] Pharmaceutical and Medical Devices Agency Website. https://www.pmda.go.jp/drugs/2015/P20150908002/670227000_22700AMX01003_A100_1.pdf. Accessed 21 Jan 2022.

[34] PMDA review report: Trabectedin Pharmaceutical and Medical Devices Agency Website. https://www.pmda.go.jp/files/000216480.pdf. Accessed 1 Aug 2022

[35] PMDA review report: Dabrafenib mesilate (Initial Approval) Pharmaceutical and Medical Devices Agency Website. https://www.pmda.go.jp/files/000233740.pdf. Accessed 1 Aug 2022

[36] PMDA review report: Osimertinib mesilate Pharmaceutical and Medical Devices Agency Website. https://www.pmda.go.jp/files/000220615.pdf. Accessed 1 Aug 2022

[37] PMDA review report: Ceritinib Pharmaceutical and Medical Devices Agency Website. https://www.pmda.go.jp/files/000221838.pdf. Accessed 1 Aug 2022

[38] PMDA review report: Pembrolizumab (Initial Approval) Pharmaceutical and Medical Devices Agency Website. https://www.pmda.go.jp/files/000226881.pdf. Accessed 1 Aug 2022

[39] PMDA review report: Afliberceot Beta [in Japanese] Pharmaceutical and Medical Devices Agency Website. https://www.pmda.go.jp/drugs/2017/P20170330001/780069000_22900AMX00524000_A100_1.pdf. Accessed 1 Aug 2022

[40] PMDA review report: Palbociclib [in Japanese] Pharmaceutical and Medical Devices Agency Website. https://www.pmda.go.jp/drugs/2017/P20170830001/671450000_22900AMX00963_A100_1.pdf. Accessed 1 Aug 2022

[41] PMDA review report: Avelumab (Initial Approval) Pharmaceutical and Medical Devices Agency Website. https://www.pmda.go.jp/files/000233296.pdf. Accessed 1 Aug 2022

[42] PMDA review report: Atezolizumab (Initial Approval) Pharmaceutical and Medical Devices Agency Website. https://www.pmda.go.jp/files/000234771.pdf. Accessed 1 Aug 2022

[43] PMDA review report: Olaparib (Initial Approval) [in Japanese] Pharmaceutical and Medical Devices Agency Website. https://www.pmda.go.jp/drugs/2018/P20180216001/670227000_23000AMX00022_A100_3.pdf. Accessed 1 Aug 2022

[44] PMDA review report: Durvalumab (Initial Approval) Pharmaceutical and Medical Devices Agency Website. https://www.pmda.go.jp/files/000238551.pdf. Accessed 21 Jan 2022.

[45] PMDA review report: Lorlatinib Pharmaceutical and Medical Devices Agency Website. https://www.pmda.go.jp/files/000228981.pdf. Accessed 1 Aug 2022

[46] PMDA review report: Ebcorfenib (Initial Approval) Pharmaceutical and Medical Devices Agency Website. https://www.pmda.go.jp/files/000240418.pdf. Accessed 1 Aug 2022

[47] PMDA review report: Binimetinib (Initial Approval) Pharmaceutical and Medical Devices Agency Website. https://www.pmda.go.jp/files/000240419.pdf. Accessed 1 Aug 2022

[48] PMDA review report: Dacomitinib Pharmaceutical and Medical Devices Agency Website. https://www.pmda.go.jp/files/000235181.pdf. Accessed 1 Aug 2022

[49] PMDA review report: Apalutamide (Initial Approval) [in Japanese] Pharmaceutical and Medical Devices Agency Website. https://www.pmda.go.jp/drugs/2019/P20190419001/800155000_23100AMX00311_A100_2.pdf. Accessed 1 Aug 2022

[50] PMDA review report: Necitumumab [in Japanese] Pharmaceutical and Medical Devices Agency Website. https://www.pmda.go.jp/drugs/2019/P20190702001/530471000_30100AMX00019_A100_1.pdf. Accessed 1 Aug 2022

[51] PMDA review report: Entrectinib Pharmaceutical and Medical Devices Agency Website. https://www.pmda.go.jp/files/000232794.pdf. Accessed 1 Aug 2022

[52] PMDA review report: Darolutamide Pharmaceutical and Medical Devices Agency Website. https://www.pmda.go.jp/files/000236125.pdf. Accessed 1 Aug 2022

[53] PMDA review report: Cabozantinib malate [in Japanese] Pharmaceutical and Medical Devices Agency Website. https://www.pmda.go.jp/drugs/2020/P20200323004/400256000_30200AMX00433_A100_1.pdf. Accessed 21 Jan 2022.

[54] PMDA review report: Tepotinib hydrochloride hydrate Pharmaceutical and Medical Devices Agency Website. https://www.pmda.go.jp/files/000236338.pdf. Accessed 1 Aug 2022

[55] PMDA review report: Irinotecan hydrochloride hydrate [in Japanese] Pharmaceutical and Medical Devices Agency Website. https://www.pmda.go.jp/drugs/2020/P20200407001/530457000_30200AMX00427_A100_2.pdf. Accessed 1 Aug 2022

[56] PMDA review report: Trastuzumab Deruxtecan (Initial Approval) Pharmaceutical and Medical Devices Agency Website. https://www.pmda.go.jp/files/000238706.pdf. Accessed 1 Aug 2022

[57] PMDA review report: Capmatinib hydrochloride hydrate Pharmaceutical and Medical Devices Agency Website. https://www.pmda.go.jp/files/000238885.pdf. Accessed 1 Aug 2022

[58] PMDA review report: Niraparib tosilate hydrate (Initial Approval) [in Japanese] Pharmaceutical and Medical Devices Agency Website. https://www.pmda.go.jp/drugs/2020/P20201008001/400256000_30200AMX00941_A100_1.pdf. Accessed 1 Aug 2022

[59] Notification: Guidelines for preparing the electronic package inserts of prescription drugs. [in Japanese] Pharmaceutical and Medical Devices Agency Website. https://www.pmda.go.jp/files/000241061.pdf. Accessed 21 Jan 2022.

[60] Smith T, Bohlke K, Lyman G, Carson K, Crawford J, Cross S (2015). Recommendations for the Use of WBC Growth Factors: American Society of Clinical Oncology Clinical Practice Guideline Update. J Clin Oncol.

[61] Guidance for Industry Clinical Pharmacology Section of Labeling for Human Prescription Drug and Biological Products Content and Format. U.S. Food and Drug Administration Center for Drug Evaluation and Research Website. https://www.fda.gov/media/74346/download. Accessed 21 Jan 2022

[62] Mulinari S, Vilhelmsson A, Ozieranski P, Bredström A. Is there evidence for the racialization of pharmaceutical regulation? Systematic comparison of new drugs approved over five years in the USA and the EU. Social Science Medicine 2021. 280(1) 10.1016/j.socscimed.2021.114049 10.1016/j.socscimed.2021.114049 34044186

[63] Labels for Irinotecan Hydrochloride Hydrate. U.S. Food and Drug Administration Center for Drug Evaluation and Research Website. https://www.accessdata.fda.gov/drugsatfda_docs/label/2015/207793lbl.pdf Accessed 21 Jan 2022.

[64] ICH guideline: E2E Pharmacovigilance Planning ICH Website. https://database.ich.org/sites/default/files/E2E_Guideline.pdf. Accessed 3 Feb 2022.

